# T_2_ * relaxometry of fetal brain structures using low‐field (0.55T) MRI

**DOI:** 10.1002/mrm.30409

**Published:** 2024-12-31

**Authors:** Kelly Payette, Alena U. Uus, Ella Kollstad, Jordina Aviles Verdera, Dario Gallo, Megan Hall, Joseph V. Hajnal, Mary A. Rutherford, Lisa Story, Jana Hutter

**Affiliations:** ^1^ Research Department of Early Life Imaging School of Biomedical Engineering and Imaging Sciences, King's College London London UK; ^2^ Biomedical Engineering Department School of Biomedical Engineering and Imaging Sciences, King's College London London UK; ^3^ Department of Women & Children's Health King's College London London UK; ^4^ Brighton and Sussex Medical School Brighton UK; ^5^ Guys and St. Thomas' NHS Foundation Trust London UK; ^6^ Smart Imaging Lab, Radiological Institute University Hospital Erlangen Erlangen Germany

**Keywords:** fetal brain development, low‐field MRI, T_2_* relaxometry

## Abstract

**Purpose:**

Human brain development during gestation is complex, as both structure and function are rapidly forming. Structural imaging methods using MRI are well developed to explore these changes, but functional imaging tools are lacking. Low‐field MRI is a promising modality to bridge this gap. The longer intrinsic T_2_* values at low field strengths increase the dynamic range and enable the quantification of individual brain regions with low T_2_* values, such as deep gray matter. This study investigates regional brain T_2_* quantification throughout the second half of gestation on low‐field 0.55T MRI.

**Methods:**

Dynamic multi‐echo gradient‐echo sequences were acquired in 135 cases at 0.55 T between 20 and 40 weeks' gestation. Automatic high‐resolution reconstruction and segmentation tools were developed, resulting in T_2_* values of seven individual anatomical brain structures for each subject. These regional brain T_2_* values were analyzed throughout gestation.

**Results:**

All regional fetal brain T_2_* values decreased throughout gestation (*p* < 0.01). Each anatomical brain structure had varying ranges and decay rates, with the cerebellum and white matter displaying the highest (nonfluid structure) values, with the maximum values between 350 and 400 ms at about 20 weeks. The brainstem and deep gray matter had the lowest range of T_2_* values, reaching values of 250 ms early in gestation. The matched volumetric assessment of the different structures demonstrated expected growth, matching current literature.

**Conclusion:**

Low‐field MRI allows for a detailed, regional T_2_* analysis of the fetal brain, with more inclusive norms to be developed due to its wider bore.

## 1 INTRODUCTION

Fetal MRI enables fascinating insights into early human development. Typically used from the second trimester, fetal MRI can visualize the complex cascade of events in the human brain throughout gestation, providing a macro‐level view of molecular process such as neuronal and glial proliferation, neuronal migration, and myelination.^1^ Clinically, it plays an important complementary role in antenatal diagnosis and monitoring. Structural T_2_‐weighted fetal brain imaging is used to diagnose and investigate pathologies such as ventriculomegaly,^2^, ^3^, ^4^ spina bifida,^5^, ^6^, ^7^, ^8^ congenital heart disease,^9^ and corpus callosum agenesis.^10^, ^11^ However, these studies are limited to structural information such as size and volume and do not provide insights into tissue characteristics. MRI can provide such information with a range of functional contrasts able to investigate specific tissue properties, and their recent application in fetal MRI has demonstrated the richness of imaging information possible in utero.^12^, ^13^, ^14^

One of the most promising examples of functional quantitative imaging is T_2_* relaxometry, which can provide an indirect measurement of in utero brain oxygenation by exploiting the blood oxygen–level dependent effect. In T_2_* relaxometry, the paramagnetic properties of deoxygenated hemoglobin cause local spin dephasing and thus a reduction in the signal and subsequent decreased T_2_* values.^15^, ^16^ A decrease in mean whole‐brain T_2_* over gestation has been demonstrated in healthy subjects.^14^, ^17^, ^18^ Furthermore, reduced mean whole‐brain T_2_* values were correlated with placental insufficiency,^17^, ^19^ heart defects,^20^, ^21^ and preterm birth.^22^ Whole‐brain T_2_* values have also been explored as a measure of cerebral oxygenation during maternal hyperoxia in cases of congenital heart disease,^21^, ^23^ and reduced mean brain T_2_* values have been observed during Braxton‐Hicks contractions (false labor).^24^

However, most of these studies have considered the fetal brain as one entity, averaging T_2_* values across the entire fetal brain,^17^, ^21^ or averaging two slices within the fetal brain.^14^, ^20^ Considering the fetal brain as a single volume does not capture the complexity of fetal brain growth and differing development rates between the individual brain structures, an aspect well recognized in structural fetal brain analysis.^25^, ^26^ As volumetric differences in fetal brain structures have been found in conditions such as ventriculomegaly,^27^ preterm birth,^28^ and spina bifida,^29^ there could potentially be changes in regional mean T_2_* values as well. So far, only a small number of studies included regional analysis of manually segmented subregions, and found regional differences in mean T_2_* values.^18^, ^19^ In addition to the potential clinical use of measuring regional fetal brain T_2_* values, fetal T_2_* relaxometry may provide further insights into tissue characteristics of brain development such as white‐matter myelination and neuronal migration.

Recent advances in postprocessing tools such as slice‐to‐volume registration (SVR) reconstructions have enabled the analysis of fine structures in anatomical MRI.^13^, ^30^, ^31^ They exploit redundancies in multiple, individually motion‐corrupted 2D acquisitions to obtain reconstructed high‐quality 3D volumes. Furthermore, the re‐emerging interest in low‐field fetal MRI^32^, ^33^, ^34^ using clinical 0.55T scanners pairs well with fetal T_2_* relaxometry, as the longer intrinsic T_2_* values at low field strengths increase the dynamic range and allow the study of brain structures with low T_2_* values such as deep gray matter.

This work aims to provide detailed regional fetal brain T_2_* values across gestation using low‐field MRI (0.55 T), paving the way for future applications in research studies and for clinical assessment. We hypothesize that low‐field fetal T_2_* relaxometry in combination with recent advances in SVR reconstruction algorithms and automatic segmentation techniques allow for high‐quality quantitative subregional fetal brain assessment.

## 2 METHODS

### 2.1 Study details and participants

Fetal MRI was acquired as part of two ethically approved studies (21/LO/0742, 22/YH/0210) between September 2022 and October 2023 at St Thomas' Hospital in London, UK, a tertiary referral center, on a clinical 0.55T scanner (MAGNETOM Free.Max; Siemens Healthcare, Erlangen, Germany) using a six‐element blanket coil and a nine‐element spine coil built into the table scanner. Inclusion criteria were a singleton pregnancy and maternal age over 18 years. Exclusion criteria were multiple pregnancies (i.e., twins), maternal age < 18 years, lack of ability to consent, weight > 200 kg, and contraindications for MRI such as metal implants and extreme claustrophobia. After informed written consent was obtained, pregnant individuals were scanned with continuous monitoring (including heart rate and blood pressure) in the head‐first supine position with frequent verbal interaction.

### 2.2 MRI examination

The protocol included both structural and functional imaging sequences, and lasted approximately 60 min, with a break offered midway through. For the structural sequences, T_2_‐weighted half‐Fourier acquisition single‐shot turbo spin echo (HASTE) sequences were acquired: six covering the whole uterus in orthogonal planes and three covering the fetal brain in radiological planes. The HASTE sequence had the following parameters: field of view (FOV) = 450 × 450 mm, resolution = 1.5 × 1.5 × 4.5 mm, repetition time (TR) = 2500 ms, and echo time (TE) = 106 ms. For the functional sequence, a dynamic multi‐echo gradient‐echo echo‐planar imaging (MEGE‐EPI) sequence was acquired in the maternal coronal orientation with the following parameters: resolution = 3 × 3 × 3 mm, FOV = 400 × 400 mm, GRAPPA = 2, TE = [42, 107, 172] ms; TR = 10 420 ms, number of dynamics = 15–30, with an acquisition time of 4–6 min. Maternal height and weight were recorded on the day of the fetal MRI scan and used to calculate body mass index (BMI).

### 2.3 Image analysis and statistical evaluation

**FIGURE 1:**
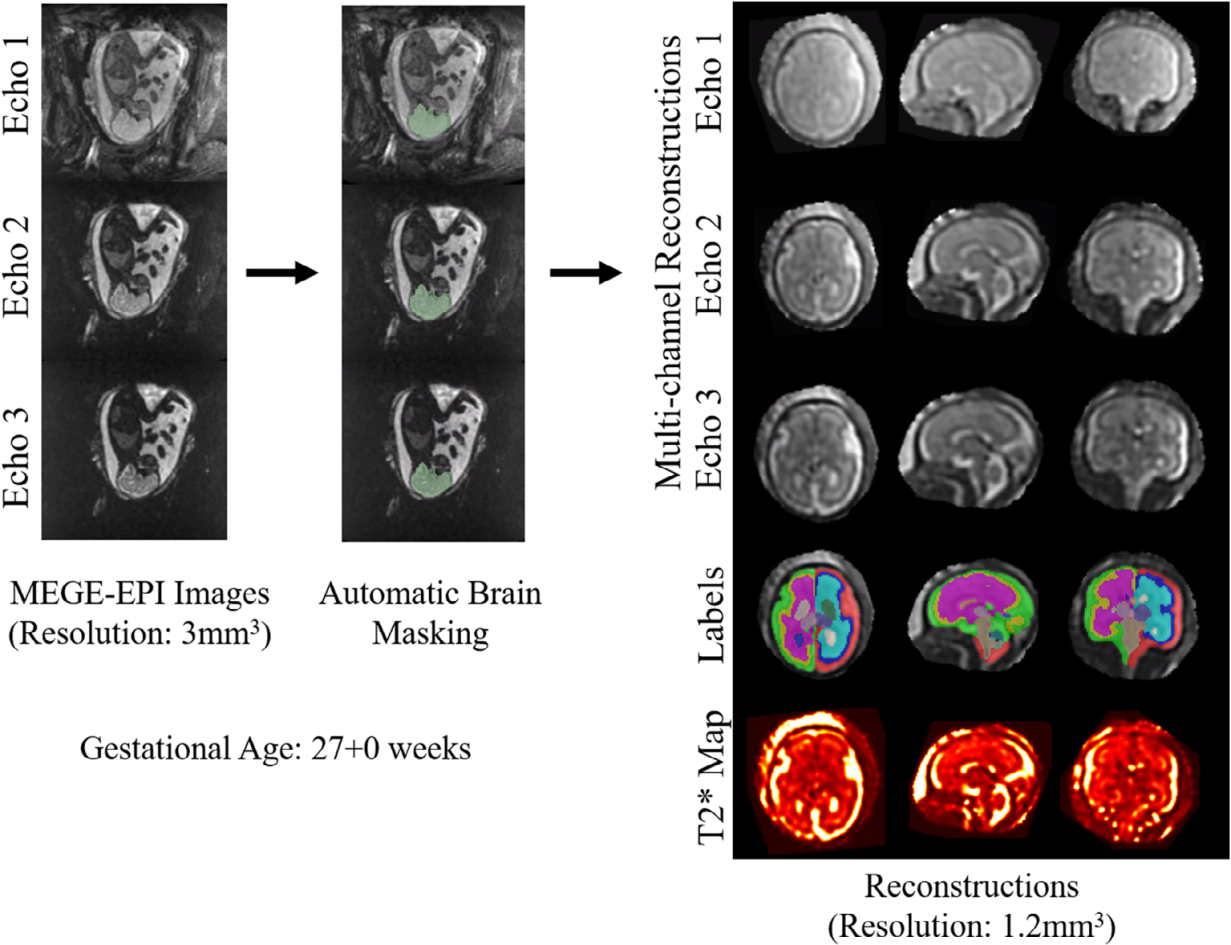
Image processing pipeline. Multi‐echo gradient‐echo echo‐planar images (MEGE‐EPI) of the whole uterus were acquired, and the fetal brain was automatically masked. Multichannel *SVRTK* reconstruction was then performed, and the reconstruction was re‐oriented to standard space, followed by automatic segmentation using *BOUNTI* and T_2_* map calculation.

Structural fetal brain reconstructions were obtained from the HASTE stacks using in‐house fully automated rigid SVR reconstructions in *SVRTK*.^30^, ^35^ Fetal brain SVR reconstructions with a resolution of 1.2 mm^3^ were created for all three echoes in the MEGE‐EPI images with multichannel *SVRTK* (including brain localization),^12^, ^13^ using all the dynamics without motion artifacts. T_2_* maps were calculated using these resulting three echo SVR reconstructions with an in‐house *Python* tool.^12^ The three‐echo SVR reconstructions were registered to the corresponding structural brain SVR reconstruction to align them and convert them into standard imaging planes.^13^ The third echo SVR reconstruction was then segmented using *BOUNTI* ^26^ into 19 different labels, which were combined into the following seven final categories: external cerebrospinal fluid, gray matter, white matter, deep gray matter, ventricles, cerebellum and vermis, and brainstem. The contrast between the white matter and cortical gray matter was most obvious in the third echo, and in the T_2_* maps, allowing for an accurate segmentation. An overview of the process can be found in Figure 1. After automatic segmentation, the labels were reviewed and corrected where necessary.

Regional fetal brain T_2_* mean values in healthy subjects were calculated, and changes across gestational age were explored in each anatomical structure. The relationship between T_2_* values and gestational age in healthy subjects was determined using a polynomial linear regression analysis using the *Python* packages sklearn, LinearRegression, and statsmodel. For subjects with a pathology, the regional fetal brain T_2_* mean values were calculated and visualized for each anatomical structure.

## 3 RESULTS

**FIGURE 2:**
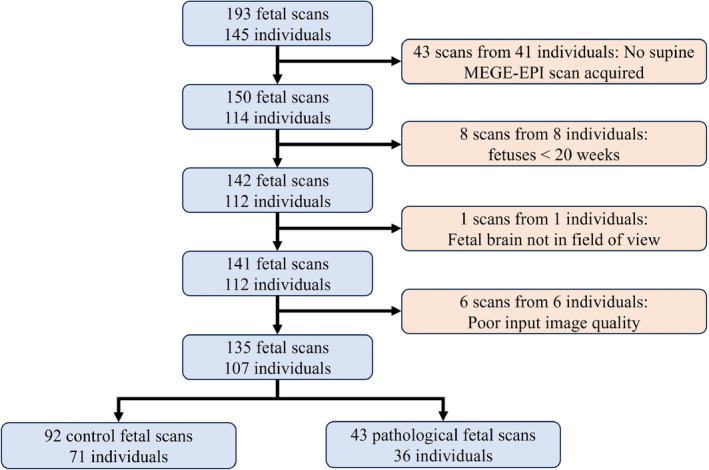
Study population flow chart.

**FIGURE 3:**
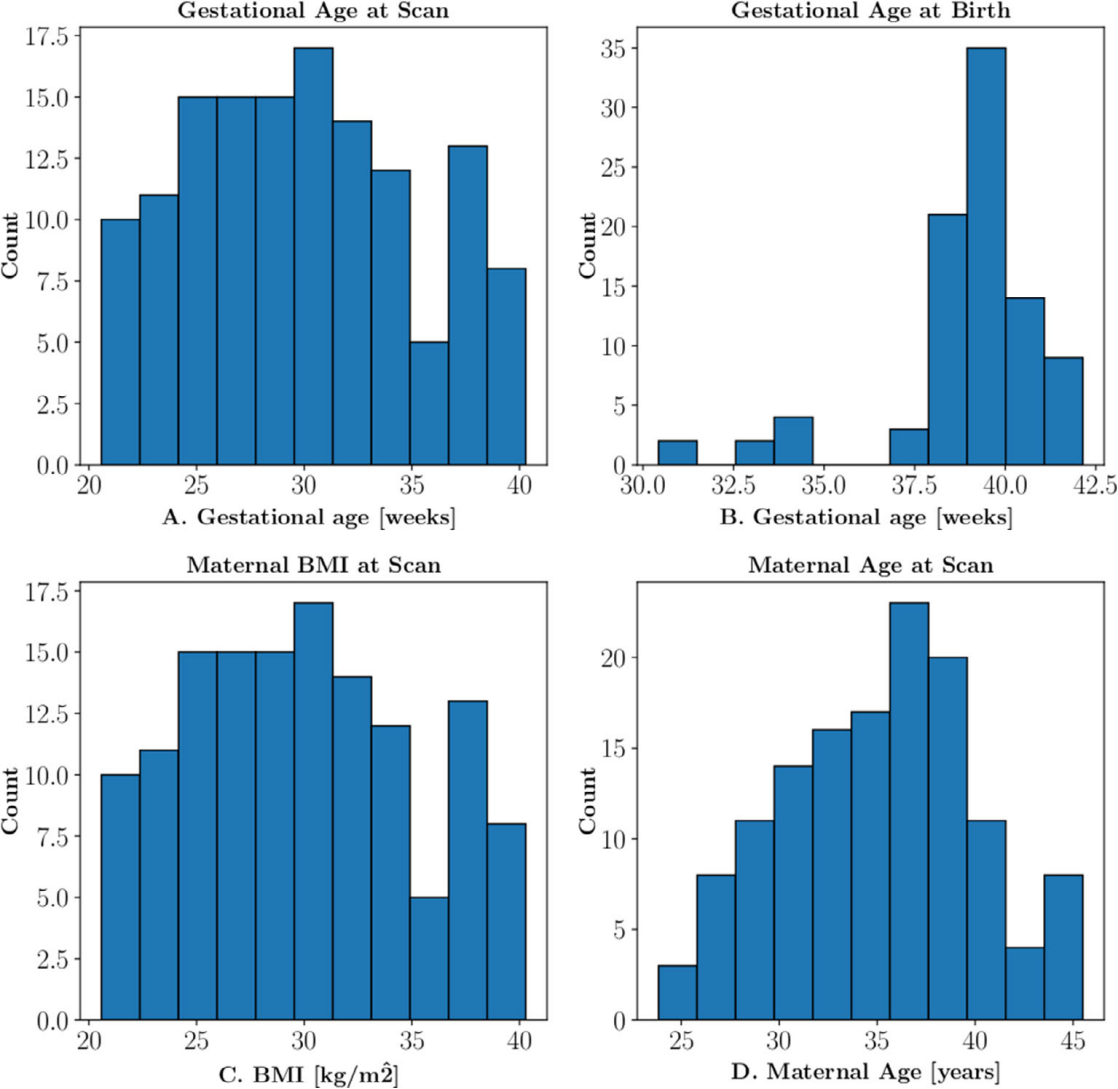
(A) Distribution of gestational age at the time of MRI scan. (B) Gestational age at birth. (C) Distribution of maternal body mass index (BMI). (D) Maternal age at the time of the MRI scan.

A total of 193 fetal research scans were performed on 145 pregnant individuals on the 0.55T scanner. Of those scans, 150 fetal scans from 114 pregnant individuals had both HASTE and MEGE‐EPI sequences. Eight further scans were excluded because the gestational age was below 20 gestational weeks: One was excluded because the brain was not included in the FOV, and 6 subjects were excluded due to excessive motion and poor image quality, leaving 135 fetal scans from 107 individuals (Figure 2). A total of 92 fetal scans (71 individuals) were acquired in healthy control subjects. Controls were determined from the outcome data after birth. Where the outcome data were not available (such as in cases where the patient delivered at another hospital), if the subject was a healthy control at the time of the MRI scan, they were assumed to have remained a control. The remaining 43 fetal scans (36 individuals) were scans with a pathology potentially impacting cerebral development. Where a subject had multiple scans taken, each scan was considered to be an independent datapoint. Demographic details of all considered cases, including details of the pathologies, can be found in Table 1, and histograms displaying details of the gestational and maternal ages scanned, as well as maternal BMI, can be found in Figure 3.

**TABLE 1:** Study population demographics.

Characteristics		*N* = 135
GA at scan (weeks)	29.9 ± 5.27	—
GA at birth (weeks)	38.9 ± 2.22 (36 individuals [45 scans] lost to follow‐up)	—
Maternal age at scan	35.0 ± 4.93	—
Maternal BMI at scan (kg/m^2^)	30.4 ± 5.64	—
Outcome	Control	93
	Ventriculomegaly	12
	Agenesis of corpus callosum	1
	Cytomegalovirus infection	2
	Hypertension (chronic, postnatal, pregnancy‐induced)	8
	Gestational diabetes	7
	Head circumference below first centile	1
	Hypoplastic left heart syndrome	1
	Preterm birth	5
	Preterm premature rupture of membranes	2
	Pre‐eclampsia	2
	Pre‐eclampsia/toxemia	2
	Raised umbilical artery Doppler	1
	Type 1 diabetes	1
	Trisomy 21	1
Ethnicity	White	82
	Mixed (white and black Caribbean, white and black African, white and Asian, other)	4
	Asian (Indian, Pakistani, Bangladeshi, other)	14
	Chinese	5
	Black (Caribbean, African, other)	11
	Any other ethnic category	7
	Unknown	12

*Note*: Some subjects may have multiple outcome categories.

Abbreviations: BMI, body mass index; GA, gestational age.

### 3.1 Regional mean brain T_2_ * throughout gestation

**FIGURE 4:**
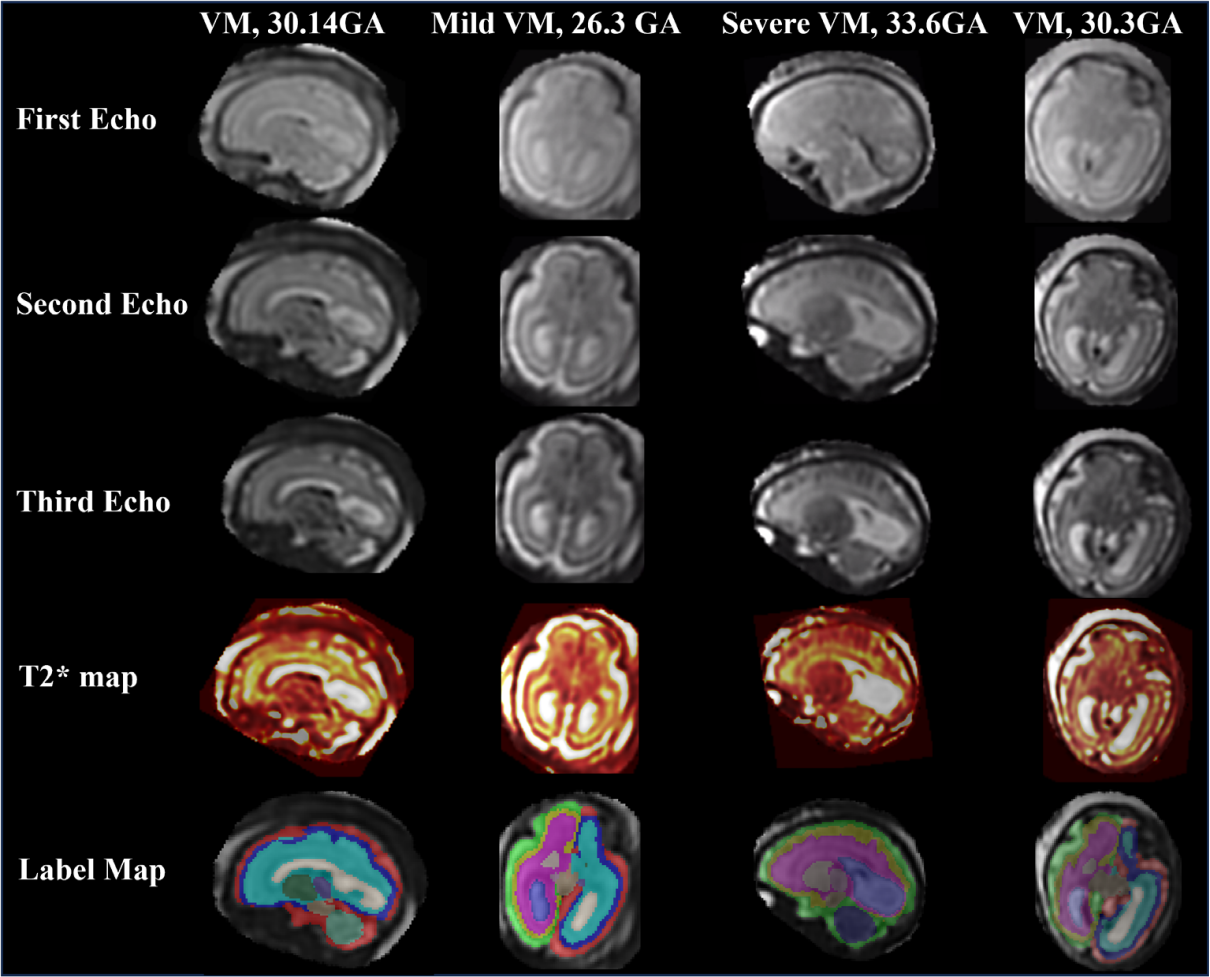
The first‐, second‐, and third‐echo brain reconstructions of fetuses with ventriculomegaly (VM), along with the T_2_* maps calculated from the three echoes and the automatically generated label maps. GA, gestational age.

**FIGURE 5:**
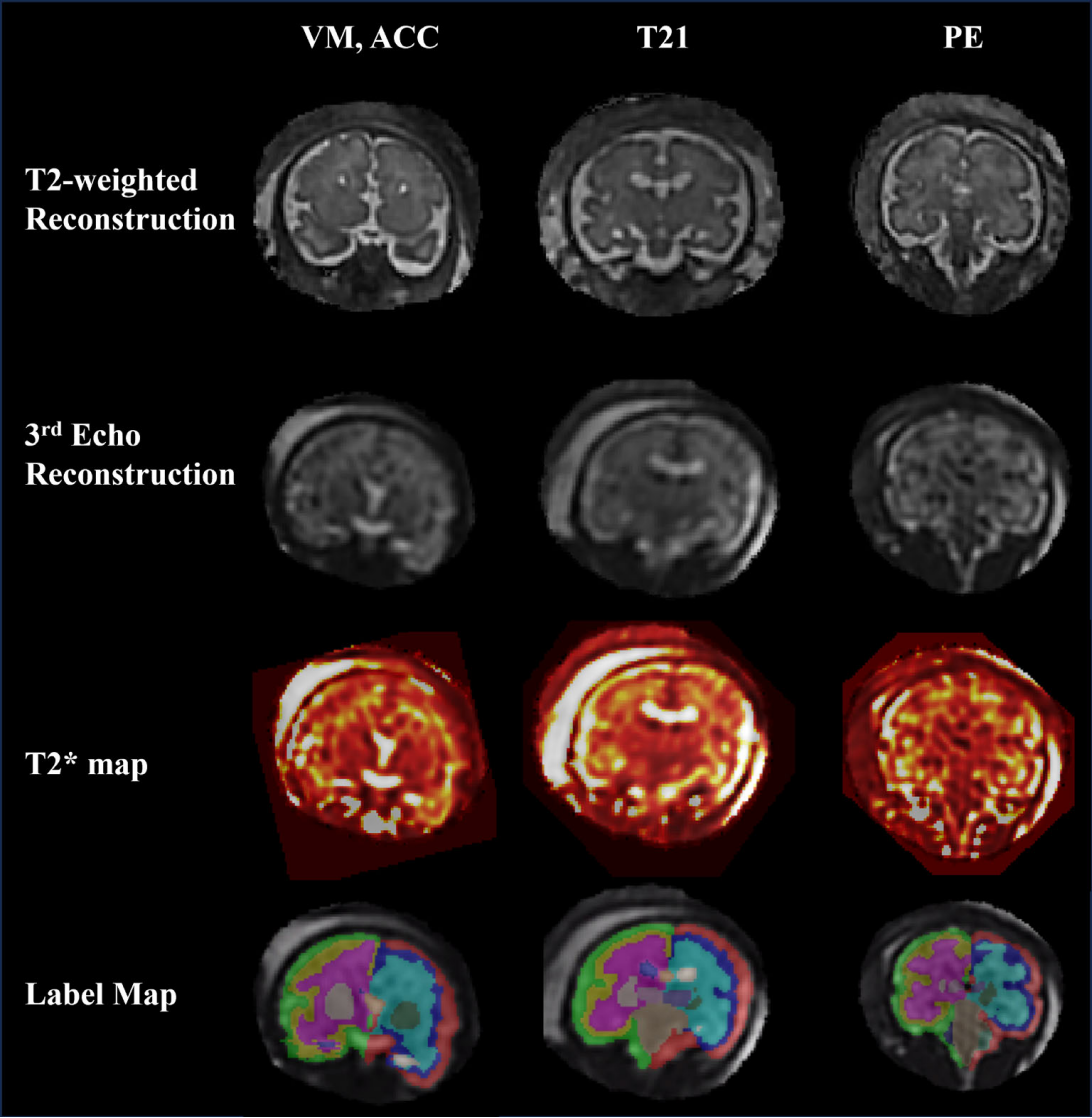
Fetal brain regional T_2_* maps for a subject with ventriculomegaly and agenesis of the corpus callosum (*left column*), Trisomy 21 (T21; *middle column*), and pre‐eclampsia (*right column*). The T_2_‐weighted reconstruction (*top row*), third‐echo reconstruction (*middle row*), and calculated T_2_* map (*bottom row*) are displayed for each subject. ACC, agenesis of the corpus callosum; PE, pre‐eclampsia; VM, ventriculomegaly.

**FIGURE 6:**
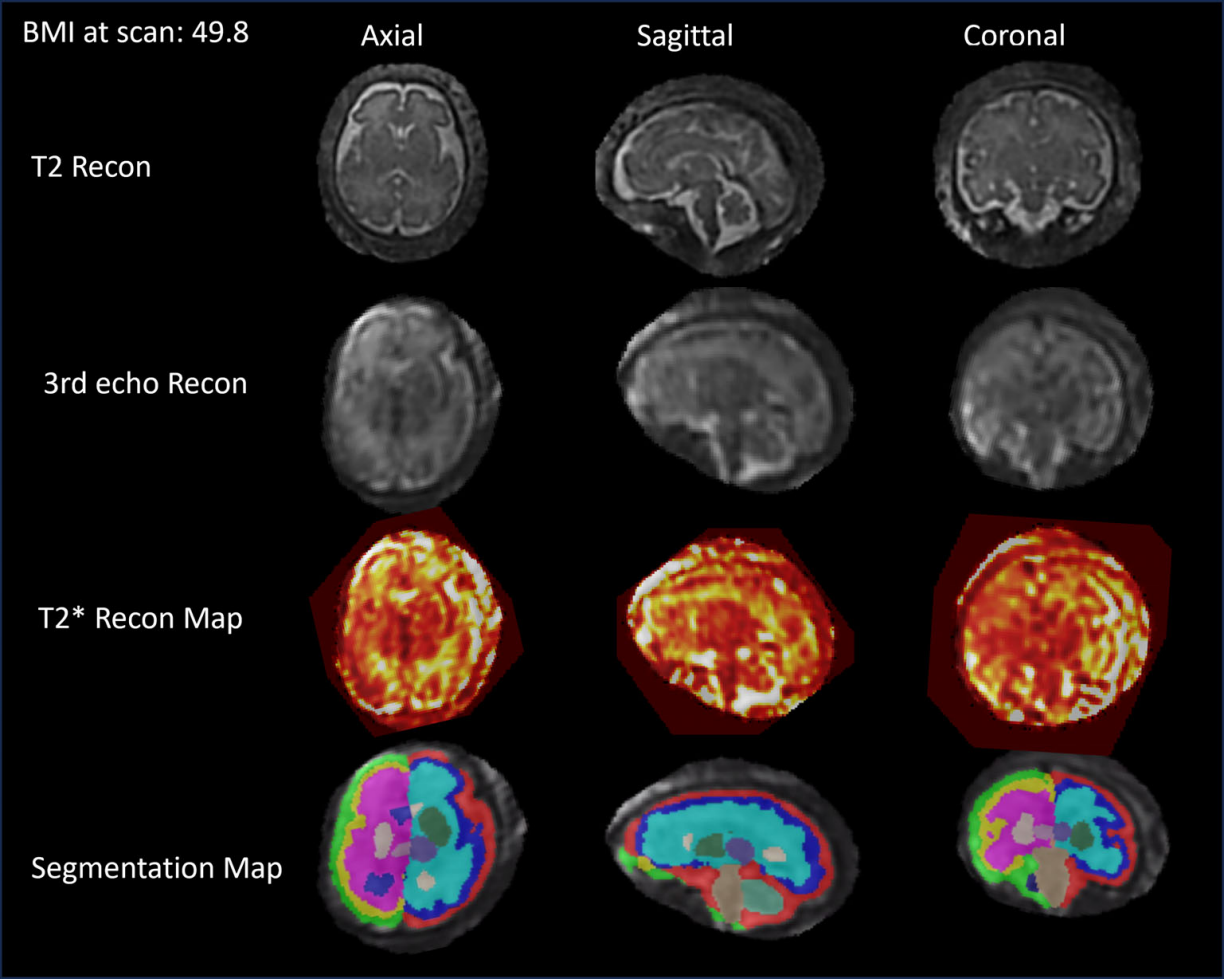
Fetal brain regional T_2_* maps for a subject with a high maternal body mass index (BMI = 49.8 kg/m^2^, 32.1 weeks' gestational age). Low‐field MRI is ideal for patients with a high BMI due to the larger bore width (80 cm, rather than the standard 60 cm), allowing for a more comfortable scanning experience.

Examples of the three‐echo SVR reconstructions, T_2_* maps, and automatic segmentations can be found in Figure 4 for subjects with ventriculomegaly. Regional brain T_2_* analysis works reliably on a variety of pathologies (Figure 5) as well as subjects with high BMI (Figure 6).

**FIGURE 7:**
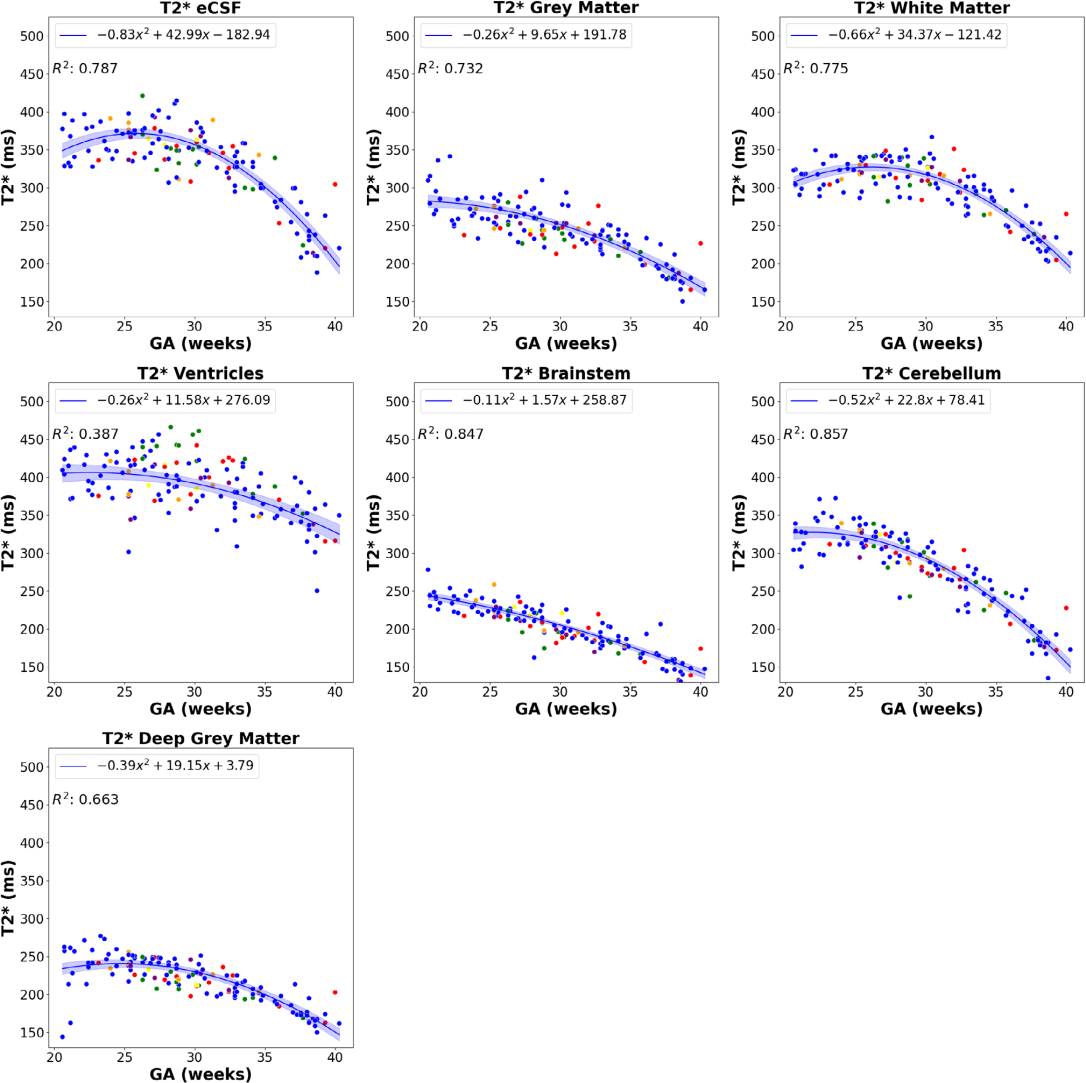
Mean T_2_* values for each anatomical brain structure across the gestational age (GA) range (external cerebrospinal fluid [eCSF; *p* < 0.01], gray matter [*p* < 0.01], white matter [*p* < 0.01], ventricles [*p* < 0.01], brainstem [*p* < 0.01], cerebellum [*p* < 0.01], deep gray matter [*p* < 0.01]). The fitting is performed only on the control fetuses. Mean T_2_* decreases in each region throughout gestation and decreases at a higher rate later in gestation for most regions except the brainstem, which appears to decrease linearly throughout gestation. *Blue*, control fetuses; *green*, ventriculomegaly; *yellow*, preterm birth; *purple*, hypertension (chronic, postnatal, pregnancy‐induced); *orange*, gestational diabetes; *red*, all other pathologies.

**FIGURE 8:**
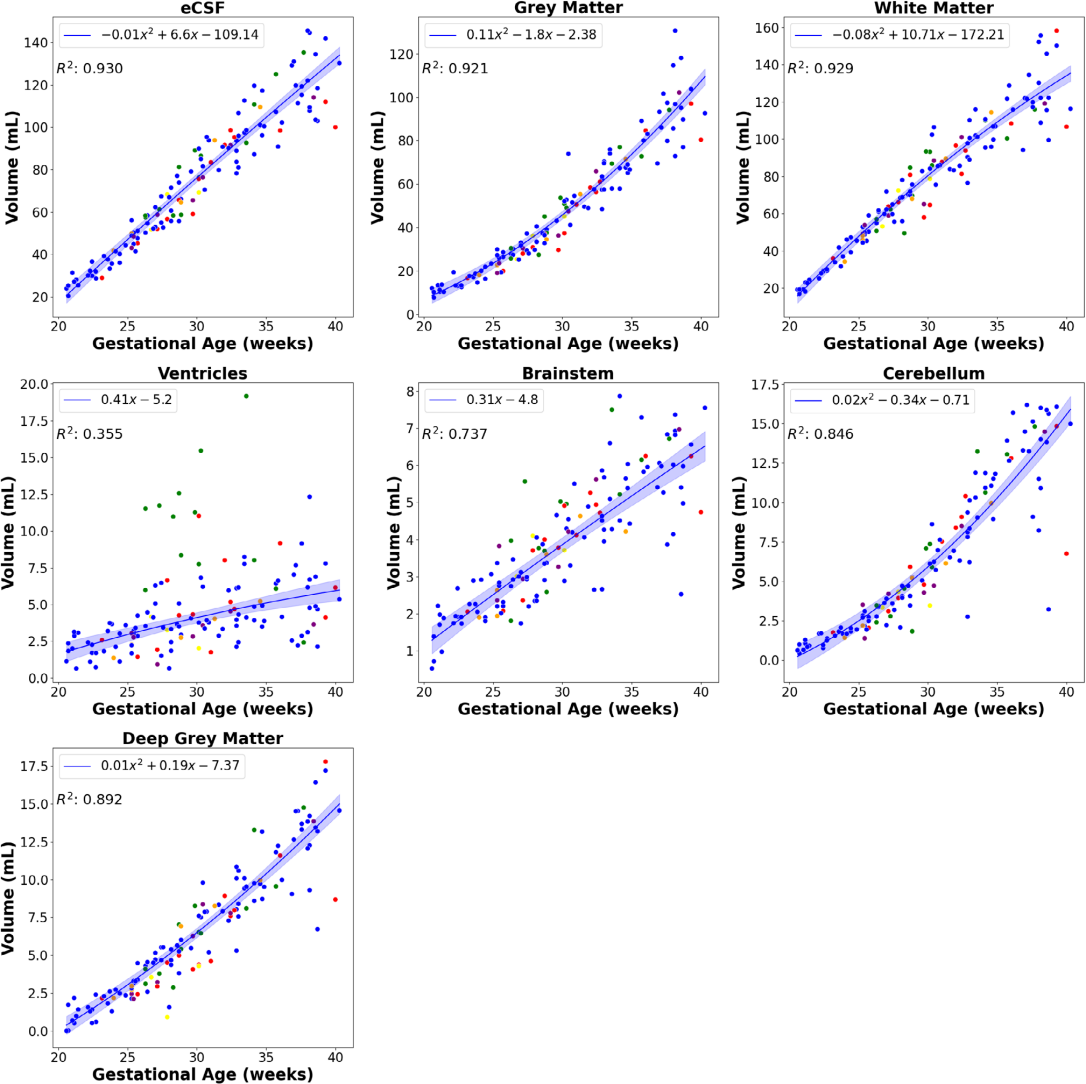
Mean volumes for each anatomical brain structure (external cerebrospinal fluid [eCSF; *p* < 0.01], gray matter [*p* < 0.01], white matter [*p* < 0.01], ventricles [*p* = 0.02], brainstem [*p* < 0.01], cerebellum [*p* < 0.01], deep gray matter [*p* < 0.01]) across the gestational age range. The fitting is performed only on the control fetuses. *Blue*, control fetuses; *green*, ventriculomegaly; *yellow*, preterm birth; *purple*, hypertension (chronic, postnatal, pregnancy‐induced); *orange*, gestational diabetes; *red*, all other pathologies.

T_2_* values across gestation for each of the seven brain regions, displayed in Figure 7, decline strongly with gestational age (*p* < 0.01 for all anatomical brain structures). As expected, all regional brain structure volumes increase significantly throughout gestation (Figure 8; *p* < 0.01 for all anatomical brain structures except ventricles, where *p* = 0.02). The absolute T_2_* values vary among anatomical brain structures, with the cerebellum and white matter displaying the highest (nonfluid) values. The brainstem and cerebellum demonstrate the strongest decay over gestation, with R^2^ values above 0.8. Detailed percentile values of all brain structures throughout gestation can be found in Table 2.

**TABLE 2:** The 5th, 50th, and 95th percentile for 2‐week periods across gestation for each of the seven brain regions in the control fetuses (*N* = 92). Gestational‐age ranges are stated in weeks + days; volumes are shown in milliliters.

Brain region		20–21 + 6 (*N* = 8)	22–23 + 6 (*N* = 10)	24–25 + 6 (*N* = 11)	26–27 + 6 (*N* = 9)	28–29 + 6 (*N* = 9)	30–31 + 6 (*N* = 8)	32–33 + 6 (*N* = 11)	34–35 + 6 (*N* = 8)	36–37 + 6 (*N* = 7)	38–40 (*N* = 11)
eCSF	Mean	357.7	362.8	365.9	370.4	358.9	357.5	331.4	307.7	266.4	228.9
	5th/50th/95th	328.3/354.1/394.3	337.0/356.1/392.4	336.4/371.8/386.7	341.9/374.2/399.1	309.3/352.5/413.2	328.9/357.1/383.4	298.8/333.5/358.5	276.6/302.0/339.6	217.3/280.9/299.3	198.7/230.7/264.0
Gray matter	Mean	294.4	272.9	266.9	258.2	259.0	254.1	233.6	225.0	201.3	178.1
	5th/50th/95th	266.8/290.3/328.7	250.1/264.5/316.6	242.1/261.0/289.0	232.4/261.7/286.5	225.9/260.1/295.1	228.1/246.0/288.1	214.9/235.7/250.6	200.4/226.9/243.0	180.3/196.4/231.0	157.7/179.4/201.7
White matter	Mean	311.7	320.5	321.6	331.6	321.0	316.9	289.7	281.3	253.0	221.7
	5th/50th/95th	293.3/317.7/323.6	292.9/320.4/346.1	290.0/329.5/340.2	308.9/341.7/343.6	286.8/333.1/346.7	280.6/311.5/359.1	264.3/294.1/315.0	249.0/286.0/301.8	228.9/249.6/290.1	203.7/219.0/243.3
Ventricles	Mean	409.0	402.6	395.9	420.6	391.1	385.1	378.5	369.0	364.4	332.9
	5th/50th/95th	371.8/412.3/438.3	379.4/398.5/429.7	324.0/415.3/436.5	372.8/427.5/453.2	364.3/390.8/412.1	343.6/387.4/418.1	325.8/380.5/416.3	347.2/358.1/402.7	340.2/358.0/397.7	275.8/336.9/372.1
Brainstem	Mean	243.0	236.7	225.6	222.3	210.0	199.6	189.2	177.6	168.7	149.6
	5th/50th/95th	227.2/241.2/267.8	221.7/236.1/252.2	217.9/223.3/238.7	211.7/222.0/237.2	175.5/215.0/229.3	187.7/196.8/220.1	176.3/184.3/205.5	163.9/177.7/188.9	145.4/165.1/202.4	135.6/148.3/160.0
Cerebellum	Mean	315.8	337.4	325.4	315.9	297.6	288.0	253.7	243.8	214.5	178.2
	5th/50th/95th	289.7/319.7/335.6	297.9/344.3/371.9	309.6/319.9/346.1	290.1/321.9/332.2	259.7/297.7/322.5	264.6/286.1/312.3	223.2/254.7/281.0	219.7/244.5/266.0	172.4/210.4/249.0	151.2/181.8/194.4
Deep gray matter	Mean	223.2	251.2	242.3	243.4	230.9	221.6	211.0	198.4	184.8	167.9
	5th/50th/95th	150.4/242.4/262.1	223.6/249.7/275.3	221.9/247.0/256.0	232.2/246.9/248.8	213.5/234.3/245.1	198.4/225.1/247.3	196.7/213.9/225.1	188.4/199.2/208.5	172.7/176.1/208.7	154.0/165.7/185.9

Abbreviation: eCSF, external cerebrospinal fluid.

## 4 DISCUSSION

Here, we successfully acquired and quantified regional fetal brain T_2_* values in the second half of pregnancy in a cohort of 135 fetal scans using low‐field (0.55T) MRI. Fetal brain T_2_* values decline significantly with gestational age, with the brainstem and cerebellum displaying the strongest decay over gestational age.

The decrease in T_2_* values over gestation agrees with other studies; however, most studies to date have primarily measured brain T_2_* values averaged over the entire brain.^14^, ^22^ One study looked at regional brain mean T_2_* values but was limited to fetuses between 20 and 28 weeks gestational age.^13^ Absolute T_2_* values vary inversely with field strength, increasing at lower field strengths. Reported 0.55T fetal brain T_2_* in a small cohort of 79 participants only considered the whole brain average.^32^ The volumes of the regions considered correspond well with the volumetric analyses conducted during pregnancy,^26^ such as 45–55 mL at 30–32 weeks in the cortical gray‐matter regions measured by high‐resolution T_2_‐weighted anatomical MRI and 51 mL (50th percentile, 30–32 gestational weeks) measured in the presented study in the same age range.

Fetal MRI plays an important complementary role to antenatal ultrasound in complicated pregnancies. Although anatomical, volumetric assessment plays the central role, functional imaging contrasts are increasingly being used to study tissue properties. However, specialist tools and long manually intensive processes often limit broader use. The presented algorithm addresses such limitations and thereby paves the way to using regional T_2_* in clinical settings. It requires no specialist planning to obtain quantitative subregional brain T_2_* values. The resulting robust normative curves from 20 to 40 weeks pave the way for wider clinical use. The wide‐bore scanner (80 cm) used in this study also allows for a more accessible MRI scan, allowing late gestation and high BMI subjects to be included, thereby creating more representative normative curves.

Altered whole‐brain T_2_* was demonstrated in a range of recent studies in major pregnancy complications such as preterm birth^22^ and pre‐eclampsia.^36^ The ability to produce regional brain T_2_* analysis could allow for an even finer, in‐depth exploration of prenatal neurological development, as T_2_* allows an (albeit indirect) measure of in utero fetal brain oxygenation. Future interesting research directions include focusing on regional brain T_2_* values in cohorts such as congenital heart disease, neural tube defects, or fetal growth restriction.

In this study, we included a large cohort of 135 scans from well‐characterized pregnancies: both healthy controls and those associated with a range of common indications for fetal MRI scans (ventriculomegaly, agenesis of the corpus callosum, Trisomy 21). The dense coverage of the control scans in the range from 20 to 40 weeks allows for the generation of reliable normative mean T_2_* curves for seven different fetal brain regions, which is a key step for wider translation. Another strength is the achieved robustness. Out of the 141 scans acquired in free‐breathing sequences without any sedation with a gestational age above 20 weeks, only six resulted in data that needed to be excluded due to motion that could not be corrected. All other scans, while exhibiting common motion, could be corrected and quantified. However, a full statistical comparison of the healthy subjects to non‐control groups was not possible due to the small numbers of individual pathologies present in the non‐control groups. Therefore, we were unable to draw conclusions from the pathologies displayed here.

A limitation of the method is the considerable proportion of the automatic segmentations that required manual correction. However, the network used was not trained for MEGE‐EPI images, but for T_2_‐weighted structural images; therefore, there is great potential that retraining will improve this network for future studies. In addition, the purpose of this study was to generate normative curves of individual brain regions; therefore, all automatic segmentations required individual validation. The fetal brain can be located in any orientation within the uterus and can therefore be in any orientation relative to the magnetic field. This study did not investigate the orientation‐dependent effects of fetal brain T_2_*, but it is an important area to explore in the future. Finally, the initial scanning protocol acquired voxels of with a resolution of 3mm^3^. Although we used SVR reconstruction to increase the resolution of the image to 1.2 mm^3^, partial volume effect is still present and affects the mean regional T_2_* values.

## 5 CONCLUSIONS

Regional fetal brain T_2_* values decrease throughout gestation as neurodevelopment progresses. Using low‐field MRI, we are able to articulate the differences between major brain anatomical structures, to create normative T_2_* curves throughout the second half of gestation. These curves have the potential to be used to help understand prenatal brain development.

## Data Availability

All presented data, including the fetal MEGE‐EPI data, the segmentations, reconstructions, and quantitative maps are available for any academic researcher upon request. The availability of such low‐field data from a significant cohort allows further development of postprocessing techniques. Similarly, all developed tools and pretrained networks are available at https://github.com/meerkat‐tools/Fetal‐T2star‐Recon.
